# Effect of dopamine on limbic network connectivity at rest in Parkinson’s disease patients with freezing of gait

**DOI:** 10.1515/tnsci-2022-0336

**Published:** 2024-05-02

**Authors:** Dione Y. L. Quek, Natasha Taylor, Moran Gilat, Simon J. G. Lewis, Kaylena A. Ehgoetz Martens

**Affiliations:** Parkinson’s Disease Research Clinic, Brain and Mind Centre, University of Sydney, Sydney, Australia; Neurorehabilitation Research Group (eNRGy), Department of Rehabilitation Sciences, KU Leuven, Leuven, Belgium; Department of Kinesiology and Health Sciences, University of Waterloo, 200 University Avenue West, Waterloo ON, N2L3G1 Canada

**Keywords:** freezing of gait, anxiety, fMRI, resting state, Parkinson’s disease, amygdala, striatum, emotion, levodopa, resting-state functional connectivity

## Abstract

**Background:**

Freezing of gait (FOG) in Parkinson’s disease (PD) has a poorly understood pathophysiology, which hinders treatment development. Recent work showed a dysfunctional fronto-striato-limbic circuitry at rest in PD freezers compared to non-freezers in the dopamine “OFF” state. While other studies found that dopaminergic replacement therapy alters functional brain organization in PD, the specific effect of dopamine medication on fronto-striato-limbic functional connectivity in freezers remains unclear.

**Objective:**

To evaluate how dopamine therapy alters resting state functional connectivity (rsFC) of the fronto-striato-limbic circuitry in PD freezers, and whether the degree of connectivity change is related to freezing severity and anxiety.

**Methods:**

Twenty-three PD FOG patients underwent MRI at rest (rsfMRI) in their clinically defined “OFF” and “ON” dopaminergic medication states. A seed-to-seed based analysis was performed between a priori defined limbic circuitry ROIs. Functional connectivity was compared between OFF and ON states. A secondary correlation analyses evaluated the relationship between Hospital Anxiety and Depression Scale (HADS)-Anxiety) and FOG Questionnaire with changes in rsFC from OFF to ON.

**Results:**

PD freezers’ OFF compared to ON showed increased functional coupling between the right hippocampus and right caudate nucleus, and between the left putamen and left posterior parietal cortex (PPC). A negative association was found between HADS-Anxiety and the rsFC change from OFF to ON between the left amygdala and left prefrontal cortex, and left putamen and left PPC.

**Conclusion:**

These findings suggest that dopaminergic medication partially modulates the frontoparietal-limbic-striatal circuitry in PD freezers, and that the influence of medication on the amygdala, may be related to clinical anxiety in freezer.

## 1 Introduction

Freezing of gait (FOG) is a common and troubling symptom of Parkinson’s disease (PD) defined as the inability to progress the feet forward despite the intention to walk [1]. It is episodic in nature and the main risk for falling in PD, thereby negatively impacting quality of life [2,3]. Understanding the phenomenology of FOG is challenging given its inconsistent and paroxysmal nature [4]. Therefore, the exact cause for FOG in PD remains poorly understood, making it a challenging symptom to manage clinically.

Several pathophysiological models have been proposed that try to explain the particular behavioural and clinical features associated with FOG [2]. For example, the interference or “cross talk” model suggests that in PD, the striatum depleted of dopamine has a reduced ability to process information concurrently across motor, cognitive, and limbic neural networks [5]. Complex gait situations can overwhelm the processing capacity of the striatum, causing the striatal output nuclei of the basal ganglia to become over-activated, sending an excess of GABAergic inhibitory projections to the brainstem locomotor control regions, which ultimately results in FOG [6]. This model posits that FOG would most likely occur during instances of increased processing demands on the striatum associated with the lack of adequate dopaminergic resources, including in the limbic circuit. Indeed, PD patients generally experience more severe FOG while performing complex gait tasks during the “off” medication state [7] and freezers tend to have worse anxiety than non-freezers [8]. Moreover, inducing anxiety via high-threat conditions while walking also resulted in more frequent FOG [8,9,10,11]. In fact, when probed with fMRI, “walking” during the high-threat condition was associated with an increase in between-network connectivity (i.e. “cross-talk”) between the motor, cognitive, and limbic circuits, in line with the cross-talk model [5,11].

Dopamine plays a central role in the cross-talk model as it strongly influences striatal processing capacity [5]. Dopamine replacement therapy is currently also the mainstay treatment for PD motor symptoms and FOG, although freezing in some patients responds poorly to dopamine therapy [12]. Given the variable effect of dopamine on anxiety and freezing [13], it would be informative to investigate how dopamine modulates cortico-striatal limbic processing in PD freezers and whether the change in network connectivity following dopamine administration is associated with freezing severity or anxiety [14].

Although several studies have used task-based and resting-state fMRI to study connectivity differences in the motor and cognitive networks in PD freezers [15,16,17,18,19,20,21], surprisingly, only one study to date has investigated limbic network differences between freezers and non-freezers which focused on their OFF medication state [22]. These findings showed an increase in resting state functional connectivity (rsFC) between the amygdala and putamen, accompanied by an increased anti-coupling between the amygdala and frontoparietal network (FPN) in PD freezers compared to non-freezers [22]. These results also correlated with overground freezing severity, suggesting that even at rest, freezers might already experience increased limbic-striatal processing load accompanied by a reduced top-down attentional control, which according to the cross-talk model might increase their propensity to freeze during actual gait. However, this study did not contrast dopaminergic state and therefore the role of dopamine on the limbic circuitry remains unclear.

To add to our understanding of the role of dopamine on the pathophysiology of freezing, the current study aimed to investigate whether dopaminergic manipulation modulates resting-state functional connectivity across the limbic-striatal and cortico-limbic networks in PD Freezers by assessing them during both the ON and OFF dopaminergic medication states. As the effectiveness of dopamine replacement therapy in alleviating symptoms of freezing and anxiety has yielded inconsistent results [13,23], the aim of the current study is also to evaluate whether the change in limbic network connectivity from OFF to ON is associated with FOG severity and degree of clinical anxiety in these patients.

## 2 Methods

### 2.1 Demographic variables

Twenty-three PD patients with FOG were recruited from the PD Research Clinic at the Brain and Mind Centre (BMC) in Sydney, Australia. All patients were diagnosed with idiopathic PD by a trained neurologist (SJGL) and FOG was confirmed through clinical observation and a positive response to item 3 of the FOG Questionnaire (FOG-Q) [24]. Patients completed a resting-state fMRI scan in both their OFF and ON dopaminergic medication states. In the OFF state, patients withdrew from their anti-Parkinson’s medication for at least 12 h (and at least 24 h if taking a dopamine agonist), and in their ON state, they were tested on their regular daily dose of anti-Parkinson’s medication. Ethical approval was obtained by the University of Sydney Human Research Ethics Committee, and all patients provided written informed consent to participate in the study. The data used in this study were retrospective data.

A trained clinician administered the Movement Disorder Society’s Unified PD Rating Scale Section III (MDS-UPDRS III) [25], as well as the Hoehn and Yahr scale [26]. A Mini-Mental State Examination (MMSE) [27] was also administered to assess general cognitive function, and all participants completed the Hospital Anxiety and Depression Scale (HADS) [28].

### 2.2 Image acquisition

The resting state brain scans were obtained at the BMC at the University of Sydney on a 3T MRI scanner (general electric) in the following order: T1 weighted anatomical images with voxel sizes of 1 × 1 × 1 mm were obtained to co-register with functional scans, and Echo planar (EPI) T2-weighted resting state functional images (repetition time, TR: 3,000 ms; echo time, TE: 32 ms; flip angle: 90°, Voxel size: 3.75 × 3.75 × 4 mm). Patients were scanned once in their ON dopaminergic medication state, and once in their clinically defined OFF dopaminergic state and the scans were retrospective. They withdrew from dopaminergic medication for at least 12 h prior to the OFF scans.

### 2.3 Image pre-processing and denoising

Pre-processing of images was completed using fMRIPrep (version 1.0.12, Nipype, 3,4 RRID:SCR_002502) [29]. T1-weighted anatomical images were corrected for intensity non-uniformity using N4BiasFieldCorrectedection (v2.1.0). Brain extraction was conducted using the ANTS toolbox [30]. The computed brain mask utilised FSL FAST to segment brain tissues into cerebrospinal fluid, grey matter, and white matter (WM). Spatial normalisation to standard space (MNI152NLin6Asym) was performed through a mutual-information based, multiscale, non-linear registration scheme with ANTs registration (v2.1.0). A custom methodology of fMRIPrep generated a reference resting state fMRI volume as well as a skull-stripped version of this volume for each individual participant. The reference image was then co-registered to the structural image with FSL’s FLIRT toolbox (v5.0.9) [31] with boundary based registration. Head motion was then estimated using FSL’s mcflirt, where head motion parameters (i.e. transformation matrices and six corresponding rotation and translation parameters) were generated [31]. Head motion parameters were calculated before time domain filtering (i.e. slice time correction). AFNI realigned all slices in time into the middle of each TR to obtain an accurate head motion estimation [32].

The resting state fMRI images were subsequently normalised into MNI space by combining all spatial transformations (head motion correction, co-registration to structural images, and normalisation to MNI space) through a one-step interpolation using Lanczos interpolation in ANTs. Using ICA’s AROMA, the identification of motion artefacts with independent component analysis was performed on the pre-processed BOLD images after removal of non-steady state volumes and spatial smoothing with an isotropic, Gaussian kernel of 6 mm full-width half-maximum [33]. Physiological noise regressors were calculated, including mean global signal, mean tissue class signal, frame-wise displacement (Nipype) [34], head motion parameters, DVARS, spike regressors temporal and anatomical principal components of most variable voxels (aCompCor, tCompCor), 6 motion parameters [35]. For more details on the FMRI-prep pre-processing pipeline, refer to https://fmriprep.org/en/stable/workflows.html.

The pre-processed rsfMRI scans then were imported into CONN v.20.b for denoising, where linear regression of potential confounding effects in the BOLD signal was conducted using Ordinary Least Squares (OLS) regression. The noise confounds were regressed out of the BOLD signal time-series included, cerebral WM, frame-wise displacement, and head motion parameters (*X*, *Y*, *Z* and Rot *X*, Rot *Y*, Rot *Z*) [35,36,37]. These nuisance regressors were selected to remove potential confounds, and for minimal denoising to be conducted to preserve important signals in the resting state fMRI data. Consequently, temporal bandpass filtering was conducted where frequencies below 0.008 Hz or above 0.09 Hz were removed from the BOLD signal. This was conducted to minimise the influence of physiological noise [38].

### 2.4 Regions of interest (ROI)

The ROIs were a priori defined based on their involvement in the cortico-limbic and limbic-striatal circuits [15,22,39]. They were selected from the Harvard Oxford atlas available in CONN and split into left and right hemispheres. ROIs included the FPN that consisted of the lateral prefrontal cortex (LPFC) and the posterior parietal cortex, the striatum (nucleus accumbens, putamen, caudate), and limbic regions (amygdala, hippocampus).

### 2.5 Functional connectivity and statistical analysis

CONN toolbox v.20.b was used to generate functional connectivity maps for each subject and each ROI using Pearson’s correlation. In the first level analysis, the ROI seeds were adjusted to fit each participant’s brain scan and functional connectivity between each pair of pre-selected ROIs. These were computed with extracted BOLD signal time courses using bivariate Pearson’s correlation measures. For each individual participant, ROI-to-ROI connectivity matrices were generated, where each pair of ROI BOLD timeseries was derived as normally distributed Fisher-transformed bivariate correlation coefficient.

These individual participants’ ROI-to-ROI connectivity maps were then combined and averaged across all participants to create an average group level connectivity matrix separately for the OFF medication scan and the ON medication scan. Each component in the matrix represents the Fisher-transformed bivariate correlation coefficient between a pair of ROI BOLD timeseries averaged across all subjects. To investigate whether dopaminergic medication altered limbic connectivity in Freezers, the rsFC between these regions (limbic to striatal ROIs, FPN to limbic ROIs, and FPN to striatal ROIs) was compared between the OFF and ON medication states in a second level within-group analysis. The rsFC difference was derived using two-tailed paired sample *t*-tests in the group-level analysis. Only connections within the same hemisphere were reported to prevent multiple corrections and false discovery rate (FDR, *α* < 0.05) corrections was applied on the resulting connections. For exploration and interpretation purposes, the OFF and ON connectivity maps derived using one-sample *t*-tests are also presented separately. As the current study focused on individual ROI-to-ROI rsFC, cluster-level inferences built into the CONN toolbox were not utilised.

Additionally, a Pearson’s correlation analysis was conducted to investigate the relationship between anxiety (HADS-Anxiety total score), freezing severity (FOG-Q Total), and changes in rsFC between OFF and ON states, where change was calculated by subtracting Freezers’ ON rsFC from OFF rsFC. We limited the secondary correlation analysis to a priori defined rsFC change scores between the amygdala and the FPN, and the amygdala and putamen, since past work has shown that these were the significant connections that differed between Freezers and non-freezers in the OFF state [22]. We also conducted correlational analysis between regions that exhibited significant differences in rsFC when OFF and ON medication states were compared, with anxiety and FOG severity scores.


**Ethical approval:** The research related to human use has been complied with all the relevant national regulations, institutional policies and in accordance with the tenets of the Helsinki Declaration, and has been approved by the authors’ institutional review board or equivalent committee. Ethical approval was obtained by the University of Sydney Human Research Ethics Committee.
**Informed consent:** Informed consent has been obtained from all individuals included in this study.

## 3 Results

### 3.1 Participant demographics

Patient demographics are detailed in Table 1. No participants satisfied MDS criteria for PD dementia [40], patients were also not on psychiatric medication, and had no major affective symptoms assessed by the DSM-V [41]. However, five participants did score above 8 on the anxiety component of the HADS, indicating mild anxiety.

**Table 1:** Participants characteristics

	Participants (mean value ± SD)
Sample (*n*)	23
Age (years)	77 ± 7.4
Disease duration	8 years 10 months
Gender (% male)	82.6% male
Neuropsychiatric examinations	
MMSE	28.2 ± 2.1
HADS depression	3.1 ± 4.72
HADS anxiety	5 ± 3.4
Motor severity	
MDS-UPDRS III (ON)	43.7 ± 16.1
H&Y	2.9 ± 0.7
FOG-Q total	11.9 ± 5.3
DDE	836.7 ± 446.8

MMSE – mini-mental state examination; HADS – hospital anxiety and depression scale; MDS-UPDRS – unified PD rating scale; H&Y – Hoehn and Yahr Scale; FOG-Q – FOG questionnaire; DDE – dopamine-dose equivalent.

### 3.2 OFF medication connectivity

#### 3.2.1 Limbic-striatum connectivity

**Figure 1:**
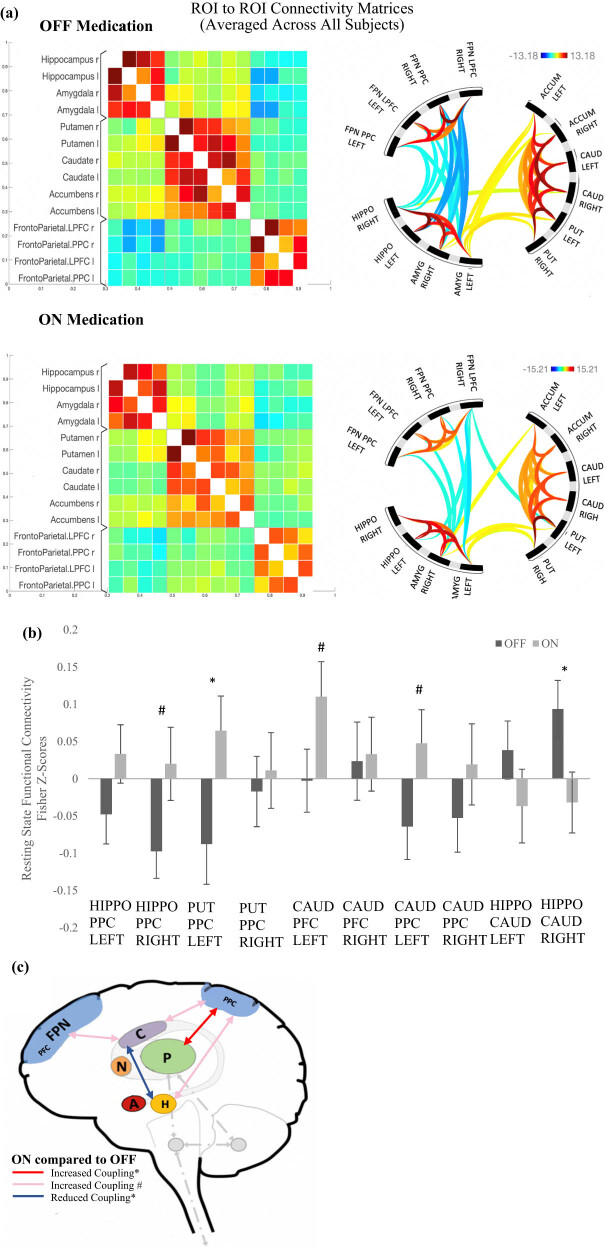
rsFC results in freezers OFF and ON dopaminergic medication. (a) ROI to ROI functional connectivity t-statistic connectivity graph and matrices of 14 ROIs averaged across all subjects between each pair of ROIs, in the OFF and ON medication states, respectively. The ROI to ROI connectivity lies on a spectrum between red and blue, where red indicates a positive rsFC and blue indicates a negative rsFC. The varying shades of colours from red to blue represents the strength of connectivity, where a darker shade indicates a stronger connectivity. (b) RSFC Fisher-Z correlation coefficient values in the OFF and ON medication states between selected ROI to ROI connectivity. Standard error bars included. Legend: (P) = Putamen; (C) = Caudate; (N) = Nucleus Accumben; (A) = Amygdala; (H) = Hippo = Hippocampus; (FPN) = Fronto-parietal Attentional Network; (FPN PPC) = FPN posterior parietal cortex. *Denotes statistical significance (*p* < 0.05, FDR Uncorrected); # Denotes a trend towards significance (*p* < 0.1). (c) This figure depicts a visual representation of the results. Darker colours represent a stronger connectivity and lighter colours represent a weaker connectivity.

In the OFF-medication state, a positive connectivity between the right hippocampus and right caudate was found (Fisher *r*–*Z* = 0.093, *p* = 0.046 FDR corrected). A positive connectivity was also found between the left amygdala and left nucleus accumbens (Fisher *r*–*Z* = 0.142, *p* = 0.004 FDR corrected), and between the bilateral amygdala and putamen (Right: Fisher *r*–*Z* = 0.134, *p* = 0.004 FDR corrected; Left: Fisher *r*–*Z* = 0.236, *p* < 0.001 FDR corrected), Figure 1a.

#### 3.2.2 Limbic-FPN connectivity

rsFC between limbic and fronto-parietal regions in the OFF medication state showed a negative connectivity between the right hippocampus and the right FPN posterior parietal cortex (PPC) (Fisher *r*–*Z* = −0.097, *p* = 0.029 FDR corrected). This negative rsFC was also observed between the right amygdala and right FPN LPFC (Fisher *r*–*Z* = −0.145, *p* < 0.001 FDR corrected) and the bilateral amygdala and FPN PPC (Right: Fisher *r*–*Z* = −0.122, *p* = 0.029 FDR corrected; Left: Fisher *r*–*Z* = −0.124, *p* = 0.037 FDR corrected), Figure 1a.

#### 3.2.3 FPN-striatum connectivity

The OFF medication state revealed a significant negative connectivity between the left putamen and left FPN LPFC (Fisher *r*–*Z* = −0.078, *p* = 0.072 FDR corrected). Other regions between the FPN and the striatum were not significantly connected.

### 3.3 ON medication connectivity

#### 3.3.1 Limbic-striatum connectivity

In contrast to what was found in the OFF state, no significant connectivity between the right hippocampus and right caudate (Fisher *r*–*Z* = −0.03, *p* = 0.549 FDR corrected), nor the left amygdala and left accumbens (Fisher *r*–*Z* = 0.100, *p* = 0.052, *p* = 0.108 FDR corrected) was found in the ON state. Similar to the OFF state, in the ON state, there also was positive connectivity between the bilateral amygdala and putamen (Right: Fisher *r*–*Z* = 0.223, *p* = 0.003 FDR corrected; Left: Fisher *r*–*Z* = 0.162, *p* = 0.001 FDR corrected), Figure 1a.

#### 3.3.2 Limbic-FPN connectivity

There was no significant connectivity between the right hippocampus and right FPN PPC in the ON state (Fisher *r*–*Z* = 0.020, *p* = 0.736 FDR corrected). Also, differing from the OFF-medication state, in the ON medication state, no significant connectivity was observed between the bilateral amygdala and FPN PPC regions (Left: Fisher *r*–Z = −0.037, *p* = 0.309 FDR corrected; Right: Fisher *r*–*Z* = −0.044, *p* = 0.408 FDR corrected). A negative connectivity was found between the right amygdala and right FPN LPFC (Fisher *r*–*Z* = –0.137, *p* = 0.010 FDR corrected) in the ON state, similar to what was observed in the OFF state.

#### 3.3.3 FPN-striatum connectivity

There was a trend toward a positive connectivity between the striatum and FPN region in the ON medication state, specifically between the left FPN LPFC and the left caudate (Fisher *r*–*Z* = 0.110, *p* = 0.064 FDR corrected), whereas these regions were not significantly connected in the OFF medication state. The significant negative connectivity between the left putamen and left FPN LPFC in the OFF state was not observed in the ON state (Fisher *r*–*Z* = 0.033, *p* = 0.549 FDR corrected).

### 3.4 Contrast between rsFMRI OFF and ON medication connectivity

To investigate whether the rsFC patterns observed in the OFF and ON medication states were significantly different from each other, we contrasted the rsFC between the two medication states as the primary comparison. Results revealed that PD patients with FOG had significantly increased positive rsFC between the right hippocampus and the right caudate in the OFF compared to the ON dopaminergic medication condition (*t*(22) = 2.88, *p* = 0.009, FDR uncorrected), Figure 1b and c. Patients also had significantly increased negative connectivity between the left putamen and left FPN PPC (*t*(22) = −2.16, *p* = 0.042, FDR uncorrected) in the OFF state compared to the ON state (Figure 1b). No significant differences in rsFC were observed between the OFF and ON medication states in other regions that were identified to have significant connectivity in the OFF medication state

#### 3.4.1 Associations between anxiety, FOG severity, and OFF–ON rsFC differences

**Figure 2:**
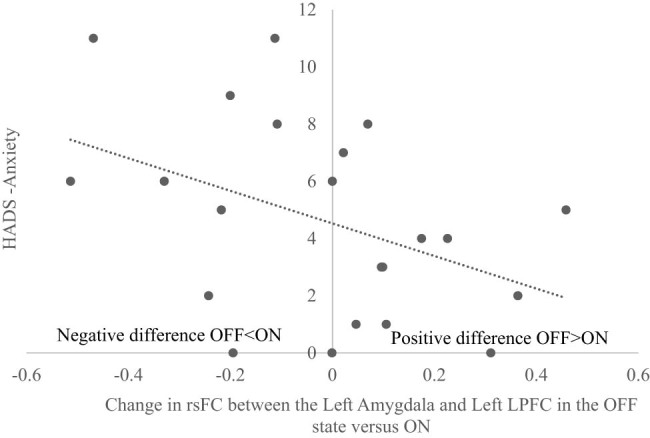
Correlation scatterplot that showed a negative correlation between HADS-Anxiety and the rsFC difference comparing OFF and ON medication states (OFF–ON) between the left amygdala and left LPFC. Findings indicated that higher baseline anxiety levels were associated with greater connectivity between these regions in the ON compared to the OFF medication state, whereas lower anxiety levels were associated with reduced connectivity between these regions in the ON compared to the OFF medication state.

A significant negative correlation was observed between HADS-Anxiety scores and the rsFC difference between the OFF and ON medication states (OFF–ON) for the left amygdala and the left FPN LPFC (*r* = −0.423, *p* = 0.050) (Figure 2). This finding indicated that freezers with higher levels of anxiety had greater anti-coupling between these regions in the OFF medication state compared to the ON state. The inverse was also observed where people with lower levels of anxiety were associated with stronger anti-coupling between these regions in the ON compared to the OFF-medication state. More anxious patients showed a greater coupling between the left amygdala and left FPN LPFC with dopaminergic medication, whilst less anxious patients showed greater anti-coupling between these regions when dopaminergic medication was administered (Figure 2). No significant connectivity was observed between HADS-Anxiety and rsFC change scores (OFF–ON) between the right amygdala and right FPN LPFC (*r* = −0.286, *p* = 0.197), left amygdala and left FPN PPC (*r* = 0.002, *p* = 0.992), right amygdala and right FPN PPC (*r* = −0.013, *p* = 0.956), left amygdala and left putamen (*r* = −0.011, *p* = 0.960), and right amygdala and right putamen (*r* = −0.236, *p* = 0.291).

**Figure 3:**
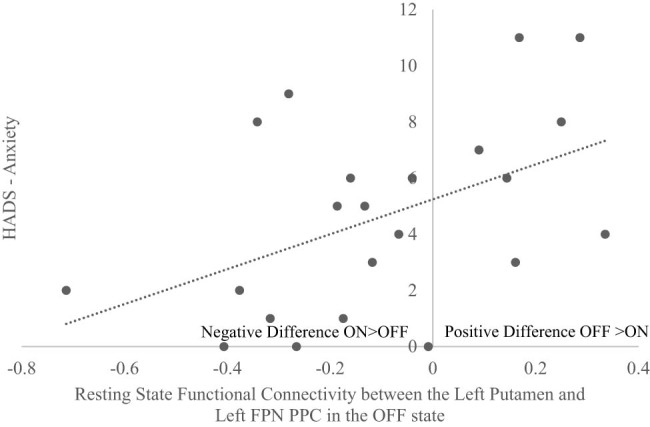
Correlation scatterplot that showed a positive correlation between HADS-Anxiety and the rsFC difference comparing OFF and ON medication states (OFF–ON) between the left putamen and left PPC. Findings indicated that higher baseline anxiety levels were associated with greater connectivity between these regions in the OFF compared to the ON medication state, whereas lower anxiety levels were associated with increased connectivity between these regions in the ON compared to the OFF-medication state.

Another significant positive correlation was observed between HADS-Anxiety scores and the rsFC difference between (OFF–ON) medication states for the Left Putamen and Left FPN PPC (*r* = 0.480, *p* = 0.024) (Figure 3). This finding indicated that freezers with higher levels of anxiety showed greater coupling between these regions in the OFF compared to ON medication state, while freezers with lower levels of anxiety showed stronger coupling between these regions in the ON compared to OFF state. This suggests that significant dopaminergic medication related rsFC changes observed between the left putamen and left FPN PPC were influenced by the level of anxiety. Freezers with more anxiety showed greater anti-coupling between the left putamen and left FPN PPC with dopaminergic medication, while less anxious freezers showed greater coupling between these regions upon being administered dopaminergic medication. In addition, a significant positive correlation was also found between HADS-Anxiety scores and the rsFC between Left Putamen and Left FPN PPC in the OFF medication state (*r* = 0.477, *p* = 0.025), this correlation was not observed in the ON medication state (*r* = −0.175, *p* = 0.437).

There were no significant correlations observed between HADS-Anxiety and the significant rsFC change scores (OFF–ON) between the right hippocampus and right caudate (*r* = 0.069, *p* = 0.760), or HADS-Anxiety and these regions in the OFF (*r* = 0.088, *p* = 0.697) or ON (*r* = 0.007, *p* = 0.975) medication states (supplementary A: Table A). With regards to FOG severity, no significant correlations were observed between FOG-Q Total scores and the rsFC difference between the OFF and ON medication states (OFF–ON) between the left amygdala and the left FPN LPFC (*r* = 0.144, *p* = 0.514), Left Putamen and Left FPN PPC (*r* = −0.275, *p* = 0.204), and right hippocampus and right putamen (*r* = −0.021, *p* = 0.924). (Supplementary A: Table B). However, a significant positive connectivity was observed between FOG-Q Total and the left amygdala and Left FPN PFC in the OFF medication state (*r* = 0.421, *p* = 0.045), this was not observed in the ON medication state (*r* = 0.245, *p* = 0.259).

## 4 Discussion

In this study, we explored the role of dopamine medication on the fronto-striatal-limbic circuit connectivity in PD freezers. The main results showed that PD freezers displayed increased rsFC between the right hippocampus and right caudate nucleus during OFF compared to ON dopaminergic medication state, while a negative rsFC was found between the left putamen and left PPC as part of the FPN. However, these findings did not survive FDR corrections and should be cautiously interpreted. A correlation was also found between the HADS anxiety scale and the rsFC difference between the medication states of the left amygdala and left LPFC of the FPN, and between HADS anxiety and the rsFC difference between the medication states of the left putamen and left FPN PPC. Together the results suggest that dopaminergic medication could have partially modulated functional connectivity between the limbic-striatal and frontoparietal-striatal networks. Specifically, differing anxiety levels resulted in differential effects with dopaminergic medication that impacted the functional connectivity between frontoparietal-striatal-limbic networks.

### 4.1 Changes in rsFC with dopaminergic medication in PD freezers

Previous studies have found that dopaminergic medication alters connectivity in sensorimotor and cognitive networks in PD patients with FOG [42,43]. However, the effect of dopamine on limbic network connectivity has not been previously investigated. One recent study found a dysfunctional fronto-parietal-striatal-limbic circuitry in PD freezers OFF their medication, where Freezers were unable to adequately recruit the FPN for top-down control over emotional processing, and hence it was postulated that an increased limbic load on the dopamine depleted striatum could lead to an increased risk of FOG events [22]. The current study went a step further to determine changes in rsFC of the fronto-striato-limbic circuitry in PD patients with FOG in their OFF and ON dopaminergic medication states. In keeping with prior work, the PD freezers in the OFF state compared to the ON state demonstrated significantly increased positive connectivity between the right hippocampus and the right caudate indicative of increased subcortical limbic load, as well as a trend towards reduced coupling between the PPC, as part of the FPN, and the hippocampus, suggestive of reduced top-down emotional control in the OFF dopaminergic state. Together these results support the notion that dopaminergic medication may reduce subcortical limbic load while at the same time improve top-down emotional control exerted by the FPN over subcortical limbic regions (i.e. hippocampus), which in turn could reduce their propensity to experience FOG in ON [22].

Indeed, a previous task-based fMRI study also showed a functional association between limbic regions and the caudate during freezing events “OFF” dopaminergic medication [44]. Moreover, a recent rsfMRI study revealed increased connectivity between the hippocampus and caudate in Freezers compared to non-freezers, where the altered nodal centralities of the right hippocampus were associated with the severity of FOG [45]. These findings illustrate that hyperconnectivity between the hippocampus and the caudate is potentially associated with the pathogenesis of FOG [45]. The current study supports and extends previous research by suggesting that dopaminergic medication could reduce limbic load on the striatum.

The current study also found increased positive rsFC between the left putamen and the left PPC of the FPN in the ON state while there was anti-coupling in the OFF state. On the one hand, the posterior cortex and subcortical systems have been associated with generation of emotional responses [46,47] and thus dopaminergic medications may facilitate top-down control over emotional responses. Alternatively, as the PPC is also implicated in sensorimotor integration [48], in particular locomotor adaptation and visuomotor control [49,50], this increased coupling could represent a non-limbic functional connection whereby the PPC is able to provide sensory input to the striatal motor circuit when the posterior striatum is adequately medicated, but not when it is depleted of dopamine.

### 4.2 Relationship between anxiety, freezing severity, and dopamine on fronto-parietal limbic circuitry

Anxiety is worse in PD freezers compared to PD non-freezers, and inducing anxiety can even trigger FOG episodes in PD [10,13,44]. Altered FPN functioning and reduced FPN-amygdala connectivity have been previously found in PD freezers and this could lead to increased limbic demands on the striatum, increasing the risk of FOG [22,46,51]. Studies have also found decreased functional connectivity between the FPN and amygdala in healthy adults with high anxiety levels and patients with anxiety disorders [52,53]. However, the interplay between anxiety and dopaminergic state in freezers has not been previously explored at the neural network level. The current study revealed a significant positive correlation between freezing severity and rsFC between the left amygdala and left PFC in the OFF medication state. This suggests that top-down control from the FPN over the amygdala in freezers in their OFF dopaminergic medication state is associated with the severity of freezing [22]. Furthermore, a negative association between the rsFC difference (OFF–ON) between the left amygdala and the left FPN LPFC and the HADS-Anxiety total score was observed. This finding indicates that in more anxious freezers, an increase in connectivity between the left FPN and the left amygdala was observed when these patients went from the OFF to the ON medication state. However, in less anxious freezers, a reduction in connectivity (increased anti-coupling) was observed between these regions comparing the OFF to ON dopaminergic medication state.

As the PFC is involved in regulating the processing of the striatum [54,55,56], during the dopamine depleted OFF medication state, freezers may not have sufficient striatal dopamine to support PFC-amygdala limbic related processing. This was observed in a previous study where an altered PFC-amygdala functional connectivity was found in freezers OFF dopaminergic medication [22]. However, when dopaminergic medication was administered in the ON state, striatal processing improves [57], for more anxious freezers, this enables the PFC to be coupled with the amygdala for limbic related processing. Whereas patients with lower anxiety levels require less top-down control of the amygdala for limbic processing, especially in the ON medication state when dopamine is restored. The results suggest that alterations to rsFC between the left amygdala and left LPFC brought about by dopaminergic medication were influenced by the degree of anxiety. As less anxious freezers might require less top-down emotional control in comparison to anxious Freezers, and this may fit the idea of freezing subtypes [13].

The existence of FOG subtypes, where situations that typically trigger the FOG phenomenon has been proposed [13]. The authors reported three phenotypes, namely, anxious, asymmetric-motor, and sensory-attention, where the subtypes represent distinctive pathological mechanisms that paroxysmally overwhelm the neural circuitry [13]. According to this explanation, the susceptibility to situations that provoke FOG should coincide with domains related to the expression of their subtypes, for instance in highly anxious freezers, limbic interference to the striatum could overwhelm striatal processing leading to FOG [13]. The current study supports this previous work, where with the administration of dopaminergic medication, more anxious freezers showed improvements in the fronto-parietal-limbic connectivity, whereas less anxious freezers displayed some deterioration in this connectivity. This finding could account for some of the heterogeneity in dopamine responsiveness in freezers. The current findings also revealed a positive association between HADS-anxiety and rsFC difference (OFF–ON) between the left putamen and left PPC. This indicates that freezers with higher anxiety levels showed reduced connectivity (increased anti-coupling) with dopaminergic medication, while less anxious freezers showed stronger connectivity in the ON state compared to the OFF. This finding further suggests that network changes associated with dopaminergic medication are not homogenous and supports the notion that different subtypes of FOG could be associated with different effects on the frontoparietal-striatal-limbic connectivity.

Research has shown that coupling between the PPC and hippocampus is also associated with emotional regulation [58,59]. Besides the significant coupling between the PPC and putamen, we similarly found marginally increased coupling between the PPC and the right hippocampus in PD freezers in ON medication and a negative connectivity in OFF. These findings should be interpreted with caution but might suggest that freezers were able to apply top-down control over emotional processing in the hippocampus only when medicated. Given that freezing is more severe in the OFF state and that the hippocampus is involved in memory retrieval [60,61], the lack of top-down control in the OFF state could result in more negative emotional processing, possibly brought upon by the worse freezing they previously experienced in this medication state, i.e. a “fear of freezing” during OFF. This interpretation remains speculative, however, as we did not assess emotional status during the rsfMRI scans.

The present study did not find any difference in amygdala connectivity between medication states. In particular dopaminergic medication did not significantly alter the dysfunctional FPN-amygdala connectivity as previously seen in PD freezers OFF their medications [22]. However, in line with prior work, the bilateral amygdala and PFC regions were significantly negatively coupled when examined in the OFF medication state, and this coupling was no longer observed in the ON medication state [22]. These findings could suggest that without adequate dopamine signalling, the FPN is rendered less capable to control bottom-up fear processing, which may in part explain exacerbated FOG under conditions of high anxiety [10,22,62]. We also found marginally increased coupling in ON between the LPFC and left caudate, which are both areas of the cortico-striatal cognitive control loop [63]. Due to their lack of motor automaticity, PD patients, and in particular freezers, are known to rely more on cognitive control during gait and movement in general [64,65]. Our finding supports the notion of a cognitive involvement in the cortico-striatal circuit of Freezers, which is already detectable during rest in ON [66,67].

### 4.3 Limitations and future directions

The current study did not obtain significant correlations between freezing severity and the difference between rsFC findings in the OFF and ON medication states. However, as the FOG-Q questionnaire was used to assess freezing severity, rather than an objective FOG assessment, this could have affected the results.

The current study investigated rsFC, where the inclusion criteria was an interval of less than 1 year between scans, this could potentially impact the veracity of our findings. However, the average interval between scans was only 2 months and 8 days, and only 8.7% of participants had an interval of more than 6 months between scans with the longest interval being 9 months and 7 days in between the two imaging sessions. Although during this time, the patient’s disease could have progressed, it should be noted that none of the patients changed their medications between the two scans. It is important to note that retrospective data were used in the study, this affected the researchers’ ability to control certain elements in the study. This includes the usage of the FOG-Q instead of the new FOG-Q and the inability to collect UPDRS scores in the OFF medication state.

Importantly, although two statistically significant changes in rsFC were observed upon the administration of dopaminergic therapy in our cohort, these findings did not survive FDR corrections. This could be due to low power in the data caused by the limited sample size in the study. The findings hence require replication in future studies with larger cohorts of PD freezers. Future studies should also further investigate whether subtypes (i.e. sensory-attention, motor, limbic) of FOG have varying dopaminergic responsiveness and characterise the associated network changes across different FOG subtypes in order to increase the understanding of the effects of dopaminergic medication on FOG. Additionally, HADS- Anxiety scores were only taken at baseline which did not enable the study to investigate the full extent of the relationship between anxiety and levodopa related changes in freezers.

Future studies could also examine the effects of other pharmacological compounds on the fronto-striato limbic circuits in PD freezers such as how anti-anxiolitic or anti-depressant therapy modulates this circuit in PD freezers, since the limbic circuit is also modulated by other neurotransmitters such as noradrenaline and serotonin [68,69]. Furthermore, a recent study that manipulated anxiety with a virtual reality threat paradigm found an increased pupil dilation when PD freezers navigated high-threat conditions in VR, thus supporting the role of noradrenaline in anxiety and FOG in PD [11]. Non-pharmacological interventions are also of interest, e.g. non-invasive repetitive transcranial magnetic stimulation and transcranial direct current stimulation have reported some improvements in the number and severity of FOG events [70,71]. Similarly, cognitive behavioural therapy, a common approach to treating state anxiety, was also shown to be effective in the treatment of anxiety in PD [72]. Future studies could also investigate if utilising such treatments of anxiety in PD could potentially aid in the reduction in FOG as well [73].

## 5 Conclusion

Dopaminergic medications may partially modulate the frontoparietal-limbic-striatal circuitry in PD freezers. In particular, it appeared that PD freezers off dopamine medication experienced increased subcortical limbic load from the hippocampus to the striatum (i.e. caudate) while at the same time having reduced top-down emotional control coming from the FPN to the striatum (i.e. putamen), a combination which may render them more vulnerable to freezing in the OFF state. Greater anxiety was also found to be associated with reduced top-down control of the FPN over the amygdala in PD freezers which was also associated with FOG severity. These findings provide further insight into the limbic contributions of freezing in PD.

## Supplementary Material

Supplementary Table
