# Supplementary material for: Effect of dopamine on limbic network connectivity at rest in Parkinson’s disease patients with freezing of gait
Source: Transl Neurosci. 2024 May 2;15(1):20220336. doi: 10.1515/tnsci-2022-0336 (PMC11066616; doi:10.1515/tnsci-2022-0336)
Supplement: Supplementary Table [file tnsci-2022-0336-sm.pdf]

# Supplementary material

**Table S1:** Pearson correlation coefficients of correlations between HADS A and regions with significant differences in resting state functional connectivity between the OFF and ON medication states (OFF–ON) obtained in the comparison analysis, and regions selected Apriori [1]

| HADS A | OFF | ON | OFF–ON |
|---|---|---|---|
| Left putamen – left FPN PPC | 0.477* | −0.175 | 0.480* |
| Right hippocampus – right caudate | 0.088 | 0.007 | 0.069 |
| Left amygdala – left FPN LPFC | −0.195 | 0.332 | −0.423* |

**Table S2:** Pearson correlation coefficients of correlations between FOGQ total and regions with significant differences in resting state functional connectivity between the OFF and ON medication states (OFF–ON) obtained in the comparison analysis, and regions selected Apriori [1]

| FOGQ total | OFF | ON | OFF–ON |
|---|---|---|---|
| Left putamen – left FPN PPC | −0.216 | 0.165 | −0.275 |
| Right hippocampus – right caudate | 0.087 | 0.105 | −0.21 |
| Left amygdala – Left FPN LPFC | 0.421* | 0.245 | 0.144 |

Note: FPN LPFC = Frontoparietal attentional network lateral pre-frontal cortex, FPN PPC = Frontoparietal attentional network posterior parietal cortex, *$p < 0.05$.

# References

[1] Gilat M, Ehgoetz Martens KA, Miranda-Domínguez O, Arpan I, Shine JM, Mancini M, et al. Dysfunctional limbic circuitry underlying freezing of gait in parkinson’s disease. Neuroscience. 2018 Mar;374:119–32
